# Blueberry bruise non-destructive detection based on hyperspectral information fusion combined with multi-strategy improved Beluga Whale Optimization algorithm

**DOI:** 10.3389/fpls.2024.1411485

**Published:** 2024-08-19

**Authors:** Xiaoxiong Sun, Liangkuan Zhu, Dayang Liu

**Affiliations:** ^1^ College of Computer and Control Engineering, Northeast Forestry University, Harbin, China; ^2^ College of Computer Science and Technology, Inner Mongolia Minzu University, Tongliao, China

**Keywords:** information fusion, feature extraction, multi-strategy, model optimization, beluga whale optimization algorithm

## Abstract

**Introduction:**

Mechanical damage significantly reduces the market value of fruits, making the early detection of such damage a critical aspect of agricultural management. This study focuses on the early detection of mechanical damage in blueberries (variety: Sapphire) through a non-destructive method.

**Methods:**

The proposed method integrates hyperspectral image fusion with a multi-strategy improved support vector machine (SVM) model. Initially, spectral features and image features were extracted from the hyperspectral information using the successive projections algorithm (SPA) and Grey Level Co-occurrence Matrix (GLCM), respectively. Different models including SVM, RF (Random Forest), and PLS-DA (Partial Least Squares Discriminant Analysis) were developed based on the extracted features. To refine the SVM model, its hyperparameters were optimized using a multi-strategy improved Beluga Whale Optimization (BWO) algorithm.

**Results:**

The SVM model, upon optimization with the multi-strategy improved BWO algorithm, demonstrated superior performance, achieving the highest classification accuracy among the models tested. The optimized SVM model achieved a classification accuracy of 95.00% on the test set.

**Discussion:**

The integration of hyperspectral image information through feature fusion proved highly efficient for the early detection of bruising in blueberries. However, the effectiveness of this technology is contingent upon specific conditions in the detection environment, such as light intensity and temperature. The high accuracy of the optimized SVM model underscores its potential utility in post-harvest assessment of blueberries for early detection of bruising. Despite these promising results, further studies are needed to validate the model under varying environmental conditions and to explore its applicability to other fruit varieties.

## 1 Introduction

Blueberries are delicious and rich in nutrients such as anthocyanins, making them popular among consumers (Yang et al., 2022). The Food and Agriculture Organization of the United Nations lists blueberries as one of the “five major health foods for humans,” while the World Health Organization considers them one of the “best fruits in terms of nutritional value.” However, blueberries are prone to mechanical damage during picking, packaging, and transportation, which can result from collisions, compression, and vibration. This mechanical damage not only affects the texture and quality of blueberries but also increases the risk of microbial contamination, shortens shelf life, and seriously impacts both the quality and economic benefits of blueberries (Zheng et al., 2022; Hou et al., 2024). Moreover, damaged tissue provides a breeding ground for pathogens, which can spread to surrounding sound fruits, further exacerbating economic losses and food safety hazards (Zheng et al., 2023). Therefore, early detection of mechanical damage in blueberries is of great significance for improving fruit quality, storage and transportation capabilities, and reducing losses in commercial value.

Hyperspectral imaging (HSI) technology combines spectroscopy and imaging techniques, capturing both spectral and spatial information simultaneously, resulting in a three-dimensional data cube comprising one spectral (wavelength) dimension and two spatial dimensions (Lu et al., 2020). Each pixel in the image contains the spectrum of the specific location. Differences in spectral reflectance enable the detection of changes in physical and chemical information before and after bruising of fruits. Spatial information enhances the accurate detection of bruised areas and sound regions in fruits. Methods integrating spectral and spatial information in HSI have been applied in the non-destructive testing of fruit quality (Gaci et al., 2023; Rajaei et al., 2024). Gao and Xu (2022) employed spectral and image analysis to predict the soluble solids content of red grapes. They employed primary and combined dimension reduction algorithms to extract the original spectral information. They also extracted image texture information using the gray-level co-occurrence matrix (GLCM). They developed a Partial Least Squares Regression (PLSR) model using spectral, image, and fused data. The findings demonstrated that the PLSR model incorporating fused information yielded superior prediction results, with correlation coefficients of 0.9775 and 0.9762 for the calibration and prediction sets, respectively. Huang et al. (2018) classified and detected varieties of deseeded cotton using HSI technology combined with image feature information. They extracted twelve morphological features such as length, width, area, and roundness from the spectral information of samples. Eleven feature bands were selected using the successive projections algorithm (SPA) as inputs combined with partial least squares discriminant analysis (PLS-DA), soft independent modeling of class analogy, k-nearest neighbor algorithm (KNN), principal component analysis combined with linear discriminant analysis, and quadratic discriminant analysis for modeling analysis. Modeling analysis using image information revealed that the overall recognition rate of the models was not high, indicating poor classification performance when relying solely on morphological features of hyperspectral images. As inputs, the spectral and morphological feature information of feature bands were fused to establish a spectrum fusion model. The results showed that the PLS-DA model with spectrum fusion had the best classification performance, with overall recognition rates of 98% and 97% for the modeling and prediction sets, respectively. Wang et al. (2023) combined HSI technology with germination tests to conduct feature correlation analysis and predict germination performance of sugar beet seeds. They extracted fourteen feature wavelengths as spectral features of sugar beet seeds using Kullback-Leibler divergence. Six image features of individual seed hyperspectral images were obtained using GLCM. PLS-DA, CatBoost, and support vector machine (SVM) with radial basis function kernel (RBF) models were established for germination prediction using spectral features, image features, and fusion features. The results demonstrated that the prediction effect of fusion features was better than that obtained using spectral features and image features. Compared with other models, the accuracy of the CatBoost model was 93.52%. The above studies all indicate that spectrum fusion models have higher detection accuracy than single-information (image or spectrum) models. However, the parameters in the models are set to default values. Sun et al. (2021) developed a spectrum feature fusion model and then optimized the model using the artificial fish swarm algorithm. The results showed that the optimized SVM model improved the detection accuracy by 2.22%, reaching 99.44%. However, meta-heuristic algorithms cannot guarantee obtaining the global optimal solution and often fall into local optimal solutions in some problems.

For the detection of mechanical damage in blueberries, Fan et al. (2017) explored the potential of hyperspectral reflectance imaging (950-1650 nm) to detect internal damage in blueberries within 30 minutes to 12 hours after mechanical impact. They developed a least squares support vector machine (LS-SVM) classification model. The results showed that the LS-SVM model established using characteristic wavelengths extracted by competitive adaptive reweighted sampling achieved overall accuracies of 93.3% and 98.0% for sound and bruised blueberries, respectively. Huang et al. (2020) investigated the potential of hyperspectral imaging in the 400-1000 nm spectral range to discriminate early diseases in blueberries. By combining the extraction of effective spectral ranges with self-scaling preprocessing methods, they developed a PLS-DA model, which achieved recognition rates of 100% and 99% for sound and early diseased blueberries, respectively. However, these studies focused on damage detection based on single (spectral) information. Relevant research on the detection of early mechanical damage in blueberries based on the fusion of spectral and image information has not been reported. Therefore, this study investigated the non-destructive detection of early mechanical damage in blueberries based on hyperspectral image information fusion.

Compared to PLS, KNN, RBF, decision tree, and random forest (RF), the SVM still achieves high classification accuracy when dealing with high-dimensional nonlinearly separable data in the presence of noise interference (Chandra and Bedi, 2021). Therefore, this study chooses SVM as the classifier for detecting blueberry bruising, optimizing the penalty coefficient *C* and kernel parameter radius *g* of SVM to improve the model’s classification accuracy. The Beluga Whale Optimization (BWO) algorithm is a novel evolutionary algorithm that simulates the foraging behavior of white whales. In the process of searching for optimal hyperparameters, the BWO utilizes cooperation and competition among individuals in the population to find the optimal solution. One of its advantages is its strong global search capability. However, compared to other evolutionary algorithms, the BWO may exhibit shortcomings in terms of convergence speed and local search capability. Based on this, this study proposes a multi-strategy improved the BWO to optimize the hyperparameters *C* and *g* of SVM.

The aim of this study is to identify bruising on blueberries using HSI technology. The specific objectives are to:

(1) Identify the optimal spectral and image feature extraction algorithms;
(2) Compare the classification accuracy of spectral fusion models with single-source (spectral or image) models to determine the best classification model;
(3) Optimize SVM using a multi-strategy improved BWO algorithm to enhance model classification accuracy;

## 2 Materials and methods

### 2.1 Sample preparation

In May 2023, a total of 800 blueberries (with diameters ranging from 12-15mm, of the variety Sapphire) were harvested from a rural area in Honghe Hani and Yi Autonomous Prefecture, Yunnan Province. They were subsequently transported to the Bioinformatics Testing Station at Northeast Forestry University. After 24 hours, 400 blueberries were randomly selected for the preparation of collision damage. The specific preparation method involved placing the samples horizontally on a test bench, with a soft rubber baffle placed on one side of the sample. A fine line directly above the sample was connected to a steel ball with a diameter of 9mm and a weight of 10g. The steel ball was raised to a 45-degree angle to the vertical plane and then immediately released to impact the sample, while recording the location of the damage. Sun et al. (2024) provided a detailed description of the preparation scheme for blueberry collision damage. Randomly split the blueberries, considering both sound and bruised specimens, into training and test sets in a ratio of 7:3.

### 2.2 HSI acquisition and correction

**Figure 1:**
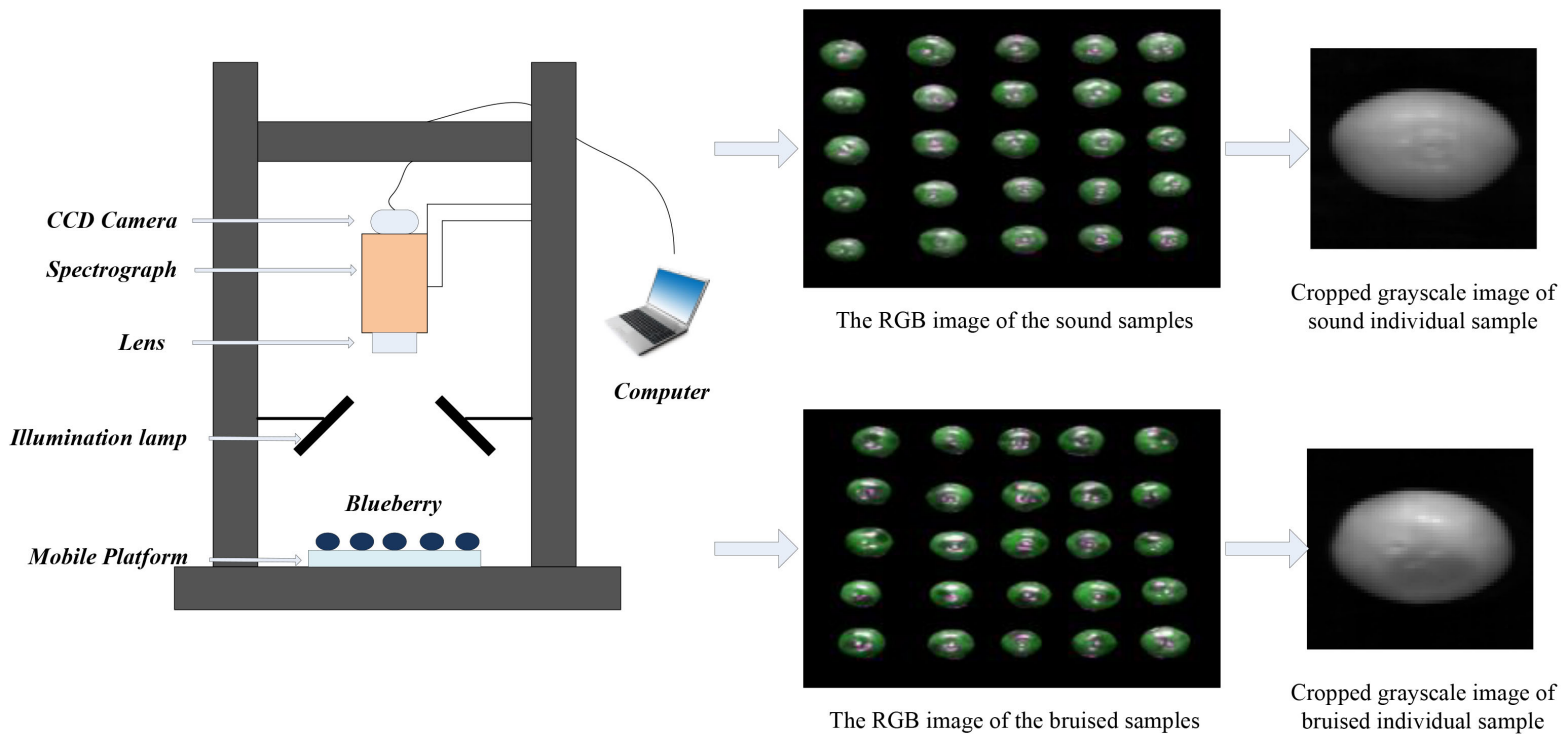
The HSI acquisition system.

The HSI system (**Figure 1**), covering a spectral range of 935nm to1720nm, involved in a spectrograph (Specim FX10, Spectral Imaging Ltd., Finland), a CCD camera (Hamamatsu, Japan) was equipped with a 34mm stationary focal lens, two illumination lamps (3900 Illuminator, Illumination Technologies, Inc. U.S.), a laptop, and a mobile platform. In order to provide a crisp image without distortion, the camera exposures period of 22 ms, and user-defined speed determined by the acquisition system was 1.6 mm/s.

During the process of hyperspectral imaging, the observed uneven illumination and noise primarily stem from the following reasons: (1) Uneven Illumination: This may occur due to unstable light source positions, variations in ambient lighting, or inherent optical irregularities in the imaging equipment. This results in varying intensities of light received across different areas of the image, leading to uneven illumination. (2) Noise: Noise can originate from electronic components within the imaging device, environmental interference, or digitization errors during signal processing. These factors introduce unwanted random signals that can degrade image quality and affect the accuracy of data analysis.

Flat Field Correction (Black-White Correction) is a commonly used technique to effectively mitigate these issues: (1) Technical Explanation: Flat Field Correction utilizes two specialized images to correct uneven illumination and noise. The “Dark Field Image” records signals produced by the imaging device under conditions of complete darkness, primarily reflecting the sensor’s inherent noise level. The “Flat Field Image” is captured under uniform illumination conditions, reflecting both the lighting and sensor response. (2) Process: During Flat Field Correction, the noise model is first extracted from the Dark Field Image and subtracted from the original image to eliminate noise effects. Then, the Flat Field Image is used to correct uneven illumination by dividing each pixel value in the original image by the corresponding pixel value in the Flat Field Image, thereby standardizing the overall image intensity.

By employing this method, Flat Field Correction significantly improves the quality of hyperspectral imaging, reducing the impact of uneven illumination and noise on data analysis, thereby enhancing the reliability and accuracy of the data.

Using the HSI system, we conducted data acquisition on samples of both sound and bruised tissues for 30 minutes (bruised area was facing the lens). We encountered uneven illumination and dark current noise during the acquisition process. Therefore, correction in black and white is required before further processing and analysis of the data. To obtain a typical white reference image, we utilized a white diffuse reflectance board with a 99% reflection efficiency. Additionally, we acquired a dark current image, known as a dark reference, to mitigate the dark current effect of the CCD detectors, as the signal of the camera chip was not zero when no light struck the detectors. The corrected image *I_c_* was obtained using Equation 1 as follow (Shicheng et al., 2021).


(1) $$I_{c}=\frac{I_{\text{R}}-I_{D}}{I_{W}-I_{D}}$$

Where *I_R_* is the captured image, *I_W_* is reference image in white and *I_D_* is reference image in dark.

When collecting samples, factors such as noise, strong light, weak light, and shadows were likely present. Therefore, hyperspectral images needed to be preprocessed to eliminate their impact on subsequent modeling efforts. The specific steps were as follows: First, the average spectral curves of sound and bruised fruit tissues from 935nm to 1720nm were analyzed, revealing significant noise in the ranges of 935nm-950nm and 1650nm-1720nm. Consequently, the 224 bands corresponding to 935nm-1720nm were reduced to 200 bands (950nm-1650nm) for analysis. Then, the spectral differences between sound and bruised tissues were compared, and the maximal difference was observed at a wavelength of 1081nm. For mask handling, the grayscale image at 1081nm was chosen. To obtain a binary image of the blueberries, a threshold segmentation algorithm was applied to the grayscale histogram to separate the blueberry image from the background. Hyperspectral imaging was then applied to mask the binarized blueberry image, thereby removing background and noise. Finally, the individual samples were cropped into images of 80 × 70 pixels.

### 2.3 Feature extraction algorithm

#### 2.3.1 Spectrum feature extraction algorithm

The SPA was a technique primarily used in chemometrics for feature selection and data compression (Soares et al., 2013). It was introduced by Haaland and Thomas in 1988. SPA aimed to extract relevant information from high-dimensional datasets by iteratively projecting the data onto lower-dimensional subspaces while preserving the variance of interest. The algorithm proceeded by identifying the variable (or feature) that contributed the least to the information of interest and removing it through successive orthogonal projections. This process was repeated until the desired dimensionality reduction was achieved or a stopping criterion was met. SPA was particularly useful in situations where the dimensionality of the dataset was high relative to the number of observations, as it helped to reduce computational complexity and improve interpretability without significant loss of relevant information. It found applications in various fields such as spectroscopy, chemometrics, and pattern recognition, where extracting meaningful features from complex datasets was crucial for analysis and interpretation.

#### 2.3.2 Image feature extraction algorithm

The GLCM served as the basis for the most widely used texture measurements (Zulpe and Pawar, 2012). According to their texture content regarding contrast, energy, entropy and homogeneity, regions in an image were characterized using texture analysis. A square matrix comprised of entries representing the relative frequency (*P_i,j_*) of occurrence of pairs of pixels with the same grey level that were separated from one another by a specific amount (D) in a specific direction (0°, 45°, 90°, or 135°) was used. The amount of emergence of the pair of grey levels *i* and *j*, which were spaced away in the image by a distance D, was represented by each item (*i,j*) in the GLCM. To represent texture, the following GLCM parameters were determined using a MATLAB program, as shown in Equations 2–5 (Benco et al., 2014):


(2) $$Contrast=\sum_{i,j=0}^{N-1}P_{i,j}{\left(i-j\right)}^{2}$$

(3) $$Energy=\sum_{i,j=0}^{N-1}{P_{i,j}}^{2}$$

(4) $$Entropy=\sum_{i,j=0}^{N-1}P_{i,j}(-InP_{i,j})$$

(5) $$Homogoneity=\sum_{i,j=0}^{N-1}\frac{P_{i,j}}{1+{\left(i-j\right)}^{2}}$$

Where the average value and standard deviations of the total of the rows and columns in the GLCM matrix are denoted by α_x_, α_y_, β_x_, and β_y_ respectively.

### 2.4 Classification model

The SVM, a method based on kernels, mapped input variables to high-dimensional feature space using kernel functions and extracted linear hyperplanes from feature space as decision functions to solve classification problems (Neumann et al., 2005). Considering its strong performance in many classification studies, the SVM was utilized in this study to model blueberry damage classification, and Gaussian radial basis functions were employed as the kernel functions.

The RF, as an ensemble learning algorithm based on decision trees, is widely used for classification and regression tasks (Rigatti, 2017). In this study, the classification models were developed by constructing multiple decision trees and intergrating their prediction outcomes.

The PLS-DA is a statistical method used primarily in classification tasks to analyze data and identify patterns or relationships between predictors (independent variables) and a categorical outcome (dependent variables) (Huang et al., 2018). It aims to maximize the separation between classes or categories in the data.

### 2.5 Optimization parameter algorithm

The BWO was a population-based metaheuristic algorithm that optimized model parameters through beluga whales swimming, foraging, and bubble-net feeding behaviors (Zhong et al., 2022). Due to its simple algorithmic structure and its excellent global search capabilities, it also demonstrated outstanding performance in various fields such as machine learning, economic load dispatch in power systems, and workshop scheduling. Since BWO was based on a population mechanism and utilized beluga whales as search agents, each beluga whale served as a candidate solution that was continuously updated during the optimization process. The position matrix modeling of the search agents was represented by Equation 6 as shown below:


(6) $$X=\left[\begin{array}{cccc} x_{1,1} & x_{1,2} & \cdots & x_{1,d}\\ x_{2,1} & x_{2,2} & \cdots & x_{2,d}\\ \vdots & \vdots & \vdots & \vdots \\ x_{n,1} & x_{n,2} & \cdots & x_{n,d} \end{array}\right]$$

Where n represents the population size of beluga whales, d is the dimension of design variables. For all beluga whales, the corresponding fitness values are stored as Equation 7.


(7) $$F_{x}=\left[\begin{array}{c} f(x_{1,1},x_{1,2},\ldots ,x_{1,d})\\ f(x_{2,1},x_{2,2},\ldots ,x_{2,d})\\ \vdots \\ f(x_{n,1},x_{n,2},\ldots ,x_{n,d}) \end{array}\right]$$

Additionally, the beluga whales transitioned from the exploration phase to the development phase through the balancing factor *B_f_*, as depicted in Equation 8 for initialization modeling.


(8) $$B_{f}=B_{0}(1-T/2T_{\max})$$

Where *B_0_* is a random number between (0,1), *T* represents the current iteration number, and *T_max_* denotes the maximum iteration number. When $B_{f}>0.5$ , the beluga whales are in the exploration phase, exhibiting mirrored swimming; when $B_{f}\leq 0.5$ , they are in the development phase, engaging in prey behavior.

#### 2.5.1 Exploration phase - swimming

During the exploration phase of BWO, they mimicked the paired swimming behavior of beluga whales, moving randomly in mirrored or synchronized fashion, as described in Equation 9.


(9) $$\left\{ \begin{array}{l} X_{i,j}^{T+1}=X_{i,p_{j}}^{T}+(X_{r,p_{1}}^{T}-X_{i,p_{j}}^{T})(1+r_{1})\sin(2\pi r_{2})\;,j=even\\ X_{i,j}^{T+1}=X_{i,p_{j}}^{T}+(X_{r,p_{1}}^{T}-X_{i,p_{j}}^{T})(1+r_{1})\cos(2\pi r_{2})\;,j=odd \end{array}\right.$$

Where *P_j_* is a random integer for dimension *d*, $X_{i,p_{j}}$ represents the value of the *i*-th whale in dimension *P_j_*, while $X_{r,p_{1}}$ represents the position of a random whale, *r_1_* and *r_2_* are both random numbers, *sin()* and *cos()* denote the orientation of mirrored whales’ fins towards the water surface, and *even* and *odd* are even and odd numbers, respectively.

#### 2.5.2 Exploitation phase – predation

The development phase of BWO emulated the foraging behavior of beluga whales. Within the beluga whale population, there was mutual communication and sharing of location information. Additionally, to enhance the algorithm’s convergence capability, the Lévy flight strategy was employed. This strategy can be represented as Equation 10.


(10) $$\begin{array}{l} X_{i}^{T+1}=r_{3}X_{best}^{T}-r_{4}X_{i}^{T}+C_{1}\cdot L_{F}\cdot (X_{r}^{T}-X_{i}^{T})\\ C_{1}=2r_{4}(1-T/T_{\max}) \end{array}$$

Where *r_3_* and *r_4_* are random numbers, *X_r_* and *X_best_* represent the positions of random whales and the best whale, respectively, and *L_F_* is the Lévy flight function, which can be represented as Equation 11.


(11) $$\begin{array}{l} L_{F}=0.05\times \frac{\mu \times \nu }{{\left|v\right|}^{1/\beta }}\\ v={\left(\frac{\Gamma (1+\beta )\times \sin(\pi \beta /2)}{\Gamma ((1+\beta )/2)\times \beta \times 2^{\left(\beta -1\right)/2}}\right)}^{1/\beta } \end{array}$$

Where μ and ν are normally distributed random numbers, β is the default constant equal to 1.5.

#### 2.5.3 Whale fall phase

If the equilibrium factor $B_{f}\leq W_{f}$ (the probability of an individual whale experiencing a whale fall), then the whale fall phase was entered. Whale fall occurred because whales were prone to predation by killer whales and human activities during migration and foraging. Dead whales sank to the seabed, pseudo-sustaining the population number thereafter. Using the individual’s position, random individual positions, and whale fall step length, new individual positions were established. This process could be represented as Equation 12.


(12) $$X_{i}^{T+1}=r_{5}X_{i}^{T}-r_{6}X_{r}^{T}+r_{7}X_{step}$$

Where *r_5_*, *r_6_* and *r_7_* are random numbers, and *X_step_* is the whale fall step length, defined as follows, as shown in Equation 13:


(13) $$\begin{array}{l} X_{step}=(\mu_{b}-l_{b})\exp(-C_{2}T/T_{\max})\\ C_{2}=2W_{f}\times N\\ W_{f}=0.1-0.05T/T_{\max} \end{array}$$

Where $\mu_{b}$ and $l_{b}$ are upper and lower boundary of variables, respectively.

The algorithm details of the BWO are shown in **Table 1**.

**Table 1:** Detailed steps of the BWO algorithm.

The BWO algorithm
**Input:** Parameters (population size, maximum iterations)
**Output:** Optimal solution
1. Initialize the population and compute fitness values, identify the best solution (P*)
2. **While** *T* ≤ *T_max_* **Do**
3. Calculate whale fall probability *W_f_* using Equation 10 and balance factor *B_f_* using Equation 8
4. **For** each beluga whale (*X_i_*) **Do**
5. **If** *B_f_* (*i*) > 0.5
6. //Exploration phase
7. Randomly generate *p_j_* (*j* = 1, 2,…, *d*) for dimensions
8. Randomly select a beluga whale *X_r_*
9. Update the position of the *i*-th beluga whale using Equation 9
10. **Else If** *B_f_* (*i*) ≤ 0.5
11. //Exploitation phase
12. Update the random jump strength *C*_1_ and compute the Levy flight function
13. Update the position of the *i*-th beluga whale using Equation 10
14. **End If**
15. Validate new positions and evaluate fitness values
16. **End For**
17. For each beluga whale (*X_i_*) **Do**
18. //Whale fall phase
19. **If** *B_f_* (*i*) ≤ *W_f_*
20. Update the step factor *C*_2_
21. Compute the step size *X_step_*
22. Update the position of the *i*-th beluga whale using Equation 12
23. validate new positions and compute fitness values
24. **End If**
25. **End For**
26. Identify the current best solution P*
27. *T*=*T*+1
28. **End While**
29. Out the optimal solution

### 2.6 Multiple-strategy improvement method

Regarding the BWO algorithm, there are issues such as slow convergence speed and susceptibility to local optima that arise when optimizing SVM model hyperparameters. This study aims to improve the following four aspects: (1) diversifying the initial population using the good point set; (2) updating the positions of the beluga whales using the elite pool strategy; (3) updating the positions of the beluga whales using a fusion of adaptive Lévy flight and spiral search strategies; (4) updating the beluga whale population using a golden sine algorithm (Golden-SA) strategy. Ultimately, a BWO algorithm with multiple-strategy improvements is developed.

The good point set represents a method for selecting points that is both uniform and efficient (Zhang and Zhang, 2001). Points acquired through the good point set exhibit a more even distribution throughout the search space in comparison to randomly selected points. In contrast to random initialization, the algorithm’s initial positions demonstrate greater uniformity, leading to accelerated convergence. The principle as shown in Equations 14 and 15, is: Let $G_{s}$ be a unit cube in s –dimensional euclidean space. If $r\in G_{s}$ , it take the form:


(14) $$P_{n}(k)=\left\{\left(\left\{r_{1}^{\left(n\right)}\cdot k\right\},\left\{r_{2}^{\left(n\right)}\cdot k\right\},\cdots ,\left\{r_{s}^{\left(n\right)}\cdot k\right\}\right),1\leq k\leq n\right\}$$

The deviation φ(n) satisfies $\varphi (n)=C(r,\epsilon )n^{-1+\epsilon }$ , where $C(r,\epsilon )n^{-1+\epsilon }$ is a constant related only to r and ϵ (where ϵ is any positive number). Then, $P_{n}(k)$ is termed a good point set, and r was a good point. $\left\{r_{s}^{\left(n\right)}\cdot k\right\}$ represents taking the decimal part, n representes the number of points, and r={2cos(2π k /p,1≤k≤s} (where p is the minimum prime satisfying (p−3)/2≥s ). It is mapped onto the search space as follows.


(15) $$x_{i}(j)=\left(ub_{j}-lb_{j}\right)\cdot \left\{r_{j}^{\left(i\right)}\cdot k\right\}+lb_{j}$$

where $ub_{j}$ and $lb_{j}$ represent the upper and lower bounds of dimension j , respectively.

To augment population diversity, the grey wolf optimization (GWO) algorithm introduced a ranking system that employed the arithmetic average of the three top-ranked wolves as the optimal position, thereby circumventing the limitations associated with relying solely on a single best individual for guidance. Inspired by GWO, this study introduced an elite pool strategy that considered the top three individuals and their weighted average as candidate elites in the elite pool (Mirjalili et al., 2014). During position updates, a random individual from the elite pool was selected as a guide, aimed at enhancing the algorithm’s capacity to escape local optima. The process can be expressed as Equations 16–18:


(16) $${\vec{D}}_{\alpha }=\left|{\vec{C}}_{1}\cdot {\vec{X}}_{\alpha }-\vec{X}\right|,{\vec{D}}_{\beta }=\left|{\vec{C}}_{2}\cdot {\vec{X}}_{\beta }-\vec{X}\right|,{\vec{D}}_{\delta }=\left|{\vec{C}}_{3}\cdot {\vec{X}}_{\delta }-\vec{X}\right|$$

(17) $${\vec{X}}_{1}={\vec{X}}_{\alpha }-{\vec{A}}_{1}\cdot ({\vec{D}}_{\alpha }),{\vec{X}}_{2}={\vec{X}}_{\beta }-{\vec{A}}_{2}\cdot ({\vec{D}}_{\beta }),{\vec{X}}_{3}={\vec{X}}_{\delta }-{\vec{A}}_{3}\cdot ({\vec{D}}_{\delta })$$

(18) $$\vec{X}(t+1)=\frac{{\vec{X}}_{1}+{\vec{X}}_{2}{\vec{+X}}_{3}}{3}$$

Where α , β , and δ represent the first three optimal solutions, t indicates the current iteration, $\vec{A}$ and $\vec{C}$ are coefficient vectors, and $\vec{X}$ is the position vector of the grey wolf.

The vectors $\vec{A}$ and $\vec{C}$ are calculated as Equations 19 and 20:


(19) $$\vec{A}=2\vec{a}\cdot {\vec{r}}_{1}-\vec{a}$$

(20) $$\vec{C}=2\cdot {\vec{r}}_{2}$$

where components of $\vec{a}$ are linearly decreased from 2 to 0 over the course of iterations, and ${\vec{r}}_{1}$ , ${\vec{r}}_{2}$ are random vectors in [0,1] .

To enhance the algorithm’s exploration ability in the solution space and enhance its convergence accuracy, this study employed an adaptive Lévy flight step size strategy (Peng and Zhang, 2022). During the early iterations, the Lévy flight had larger step sizes, allowing for comprehensive exploration of the solution space. In subsequent iterations, the Lévy flight step sizes decreased progressively, shifting towards more refined exploration. Assuming the current iteration number is t , the maximum iteration number is T , and the position of the individual is $x_{i}$ . The update of the new position $x_{i}^{\left(new\right)}$ can be represented by Equation 21:


(21) $$\text{where }\begin{array}{l} x_{i}^{\left(new\right)}=x_{i}+\alpha (t)\cdot \frac{\mu \cdot \sigma_{\mu }}{{\left|\upsilon \right|}^{1/\beta }}\\ \alpha (t)=\alpha_{0}\cdot (1-\frac{t}{T}{)}^{\gamma }\\ \mu :N(0,\sigma_{\mu }^{2})\\ \upsilon :N(0,\sigma_{\upsilon }^{2})\\ \sigma_{\mu }={\left(\frac{\Gamma (1+\beta )\sin(\pi \beta /2)}{\Gamma (\left(1+\beta \right)/2)\cdot \beta \cdot 2^{\left(\beta -1\right)/2}}\right)}^{1/\beta }\\ \sigma_{\upsilon }=1 \end{array}$$

where $\alpha_{0}$ is the initial scaling factor, γ is the parameter controlling the step size reduction speed, set to 1 or 2, and β is the exponent parameter of the Lévy distribution, ranging (1,3] .

The spiral strategy was proposed based on the adjustment of movement distance for each position update, following a spiral shape, between the target position (optimal position) and the individual’s position when whales search for prey in the whale optimization algorithm (Mirjalili and Lewis, 2016). This strategy greatly utilized regional information, thereby improving search capability, and consequently enhancing the algorithm’s rigor and accuracy in local space development. The process can be expressed as Equations 22 and 23:


(22) $${\vec{D}}^{*}=\left|{\vec{C}}^{*}\cdot {\vec{X}}^{*}(t)-\vec{X}(t)\right|$$

(23) $$\vec{X}(t+1)={\vec{X}}^{*}(t)-{\vec{A}}^{*}\cdot {\vec{D}}^{*}$$

where t indicates the current iteration, ${\vec{A}}^{*}$ and ${\vec{C}}^{*}$ are coefficient vectors, and ${\vec{X}}^{*}$ is the position vector of the best solution obtained so far, $\vec{X}$ is the position vector. It is worth mentioning here that ${\vec{X}}^{*}$ should be updated in each iteration if there is a better solution.

The vectors ${\vec{A}}^{*}$ and ${\vec{C}}^{*}$ are calculated as Equations 24 and 25:


(24) $${\vec{A}}^{*}=2{\vec{a}}^{*}\cdot {\vec{r}}^{*}-{\vec{a}}^{*}$$

(25) $${\vec{C}}^{*}=2\cdot {\vec{r}}^{*}$$

where components of ${\vec{a}}^{*}$ are linearly decreased from 2 to 0 over the course of iterations, and ${\vec{r}}^{*}$ are random vectors in [0,1] .

The Golden-SA algorithm was based on the relationship between the sine function and the unit circle, allowing it to traverse all points on the sine function and thus all points on the unit circle (Tanyildizi and Demir, 2017). The algorithm possessed strong global search capabilities. Therefore, this study employed Golden-SA to update the white whale population, improving BWO’s global search capability, and accelerating the convergence speed of the algorithm. The process can be expressed as Equation 26:


(26) $$V_{\left(i,j\right)}=V_{\left(i,j\right)}\cdot \left|\sin(r_{1})\right|-r_{2}\cdot \sin(r_{1})\cdot \left|x_{1}\cdot D_{\left(j\right)}-x_{2}\cdot V_{\left(i,j\right)}\right|$$

where $V_{\left(i,j\right)}$ is the value of current solution in *i*-th dimension. *D* is the determined target value. $r_{1}$ is a random number in the range [0,2π] . $r_{2}$ is a random number in the range [0,π] . $x_{1}$ and $x_{2}$ are the coefficients obtained by the golden section method.

The algorithm details of the multi-strategy improved BWO are shown in **Table 2**.

**Table 2:** Detailed steps of the multi-strategy improved BWO algorithm.

The multi-strategy improved BWO algorithm
**Input:** Parameters (population size, maximum iterations)
**Output:** Optimal solution
1. Initialize the population using a good point set for diversification and compute fitness values, identify the best solution (P*)
2. Create and update the elite pool based on the initial population
3. **While** *T* ≤ *T_max_* **Do**
4. Calculate whale fall probability *W_f_* using Equation 10 and balance factor *B_f_* using Equation 8
5. **For** each beluga whale (*X_i_*) **Do**
6. **If** *B_f_* (*i*) > 0.5
7. //Exploration phase
8. Randomly generate *p_j_* (*j* = 1, 2,…, *d*) for dimensions
9. Randomly select a beluga whale *X_r_*
10. Update the position of the *i*-th beluga whale using Equation 9
11. **Else If** *B_f_* (*i*) ≤ 0.5
12. //Exploitation phase
13. Update the random jump strength *C*_1_ and compute the adaptive Lévy flight and spiral search strategies
14. Update the position of the *i*-th beluga whale using Equation 10
15. **End If**
16. Validate new positions and evaluate fitness values
17. **End For**
18. For each beluga whale (*X_i_*) **Do**
19. //Whale fall phase
20. **If** *B_f_* (*i*) ≤ *W_f_*
21. Update the step factor *C*_2_
22. Compute the step size *X_step_*
23. Update the position of the *i*-th beluga whale using Equation 12
24. validate new positions and compute fitness values
25. **End If**
26. **End For**
27. Identify the current best solution P* and update the elite pool
28. If in whale fall stage then
29. Update whale population using the golden sine algorithm
30. End If
31. *T*=*T*+1
32. **End While**
33. Out the optimal solution

### 2.7 Graphic abstract

**Figure 2:**
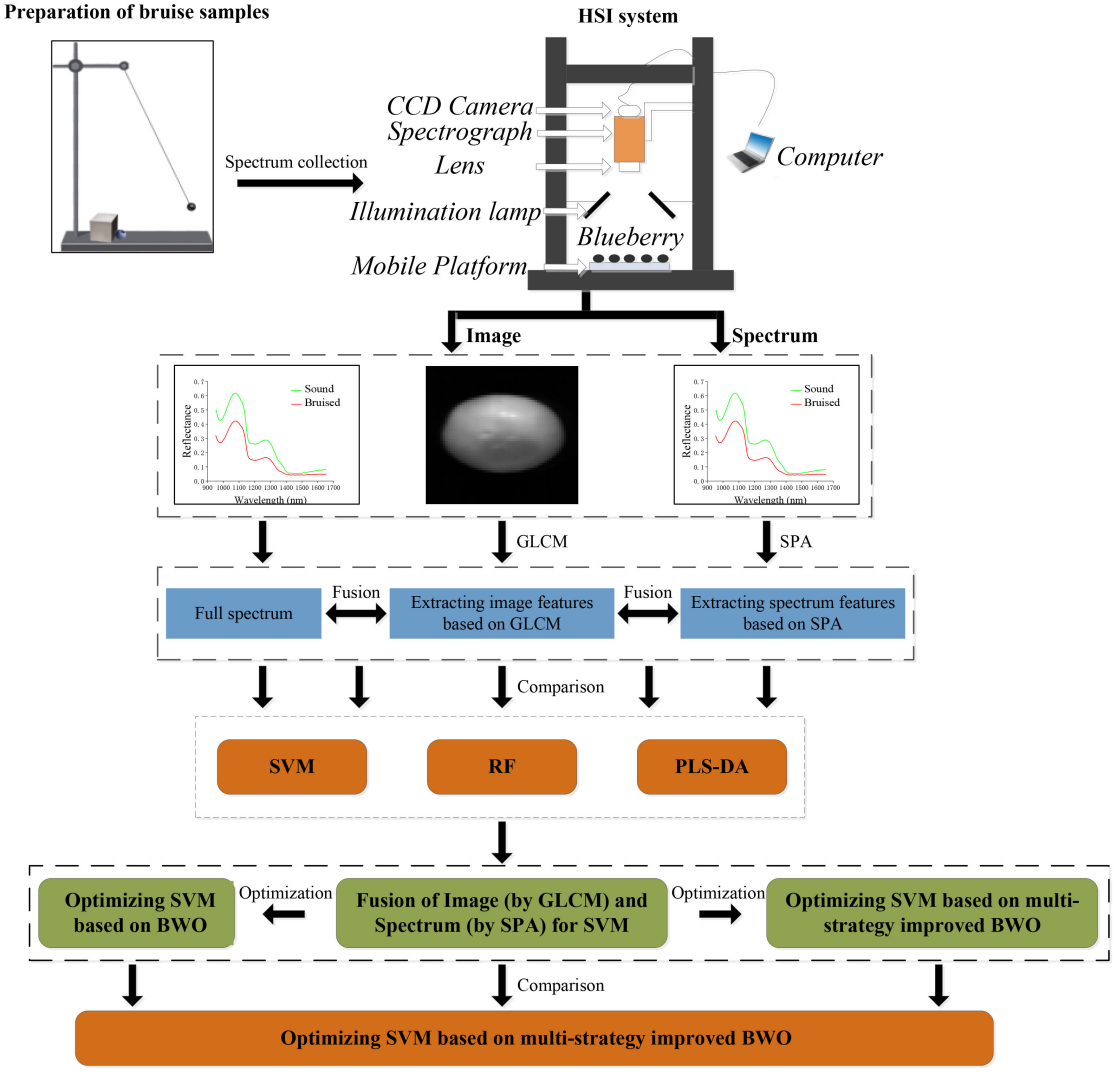
The graphic abstract of this study.

The graphic abstract of this study was presented as shown in **Figure 2**.

## 3 Results

### 3.1 Region of interest analysis

**Figure 3:**
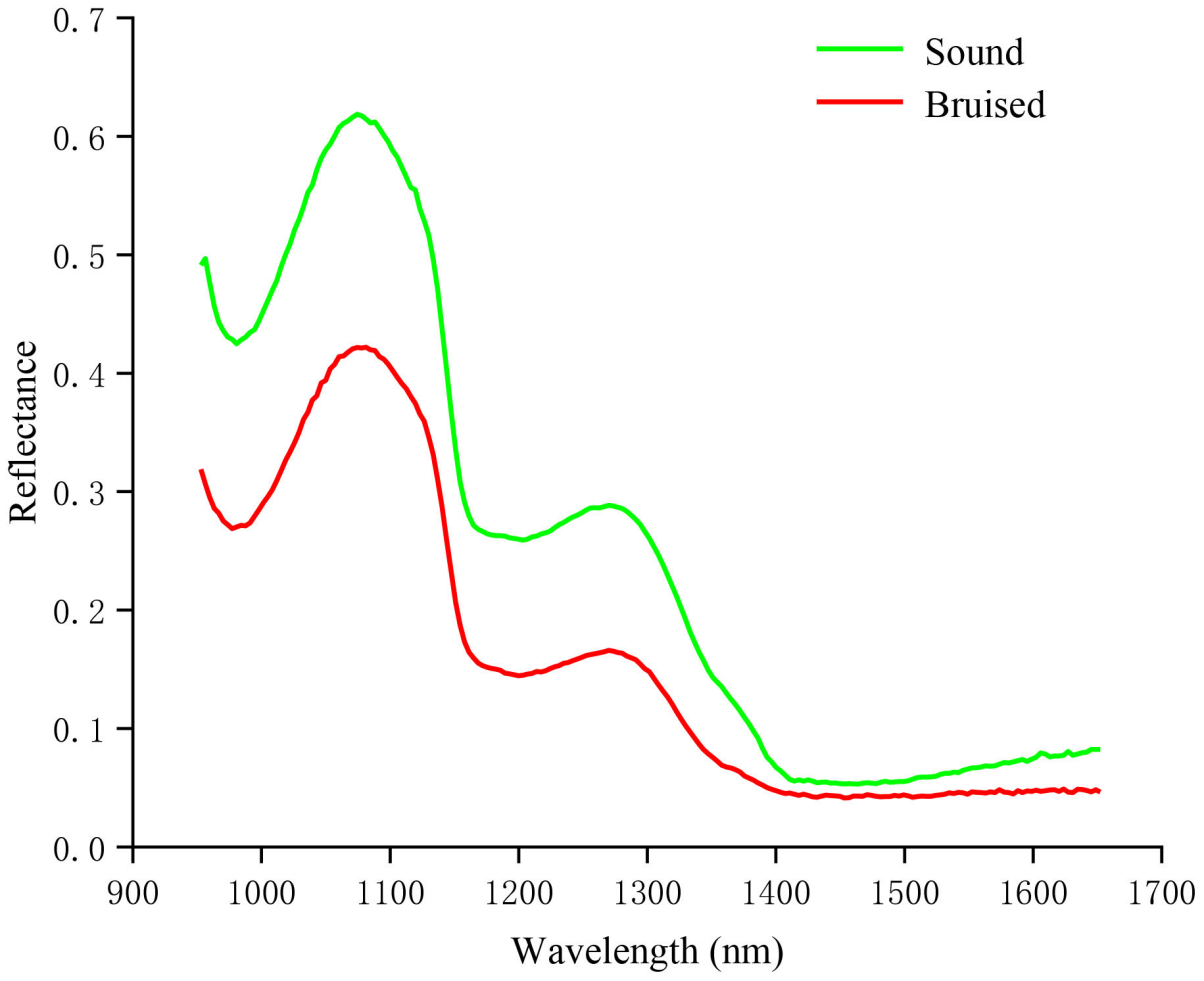
Average relative reflectance spectral curves.

**Figure 3** depicted the average relative reflectance spectral curves of sound and bruised blueberries. The HSI technique provided accurate and reliable spectra for the same biological traits, resulting in similar spectral curve trends of sound and bruised blueberries. The relative intensity of sound samples was higher than that of bruised samples. This difference may be attributed to ruptures in the cell walls and membranes of the bruised samples, which triggered oxidation and enzyme catalysis reactions, subsequently leading to a decrease in light reflection intensity due to the loss of intracellular water. Observations of absorption peaks and absorption valleys were made at 980nm, 1081nm, 1206nm, and 1280nm. The absorption peaks at 980nm and 1206nm might be related to the absorption of specific energy by hydrogen bonds in water molecules, inducing vibrational and combination vibrations (Gao et al., 2019). The absorption valleys at 1081nm and 1280nm might be related to changes in the levels of anthocyanins and carotenoids in the fruit (Amanah et al., 2021).

**Figure 4:**
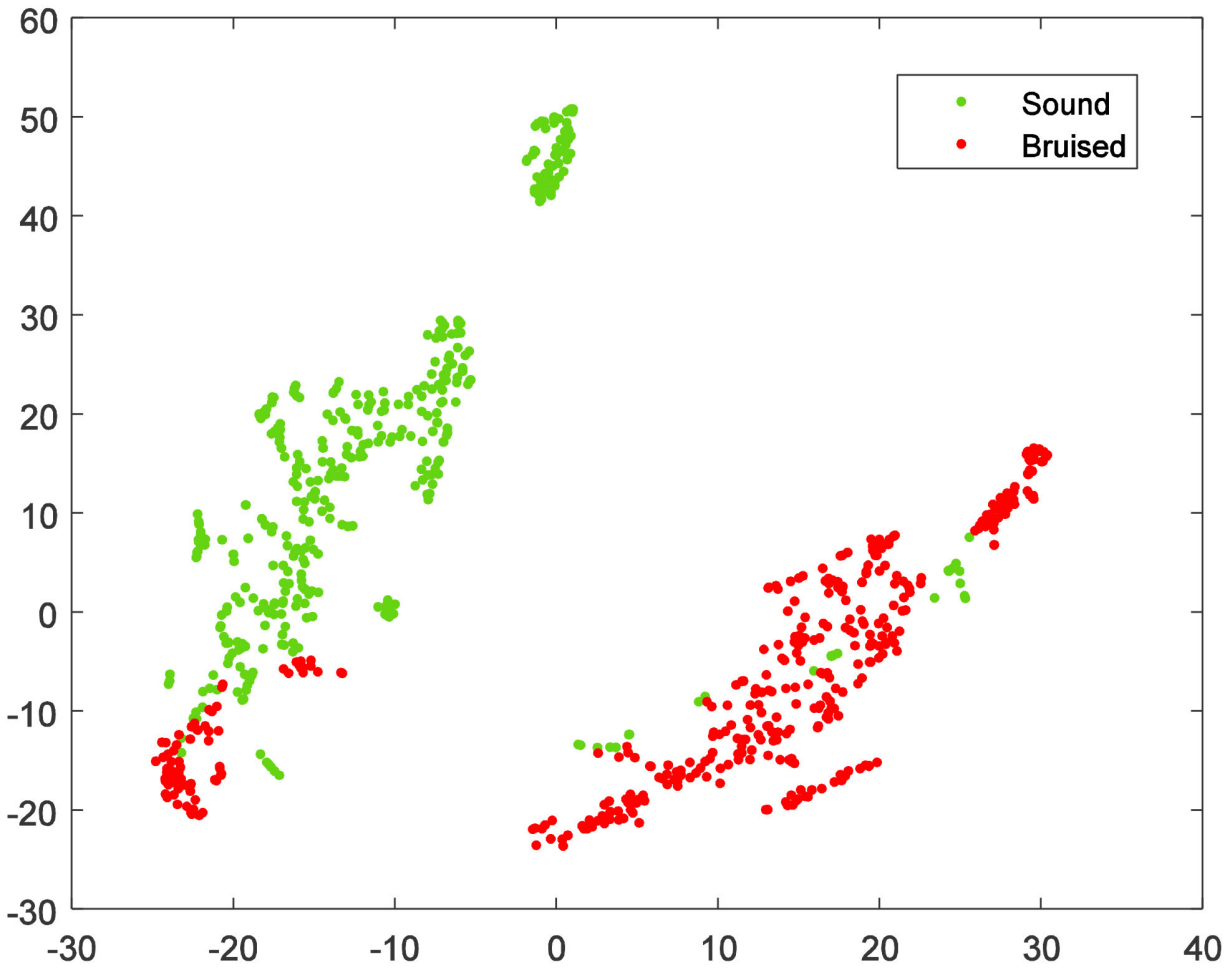
The visualization results of the t-SNE algorithm.

To visually illustrate the significant differences in reflectance spectra between sound and bruised tissues, the 200-dimensional spectral features were projected into a two-dimensional space using the t-distribution stochastic neighbor embedding (t-SNE) algorithm. The visualization results are depicted in **Figure 4**. The results in **Figure 4** demonstrate that effectively classifying sound and bruised fruits remained challenging.

### 3.2 Spectral feature analysis

**Figure 5:**
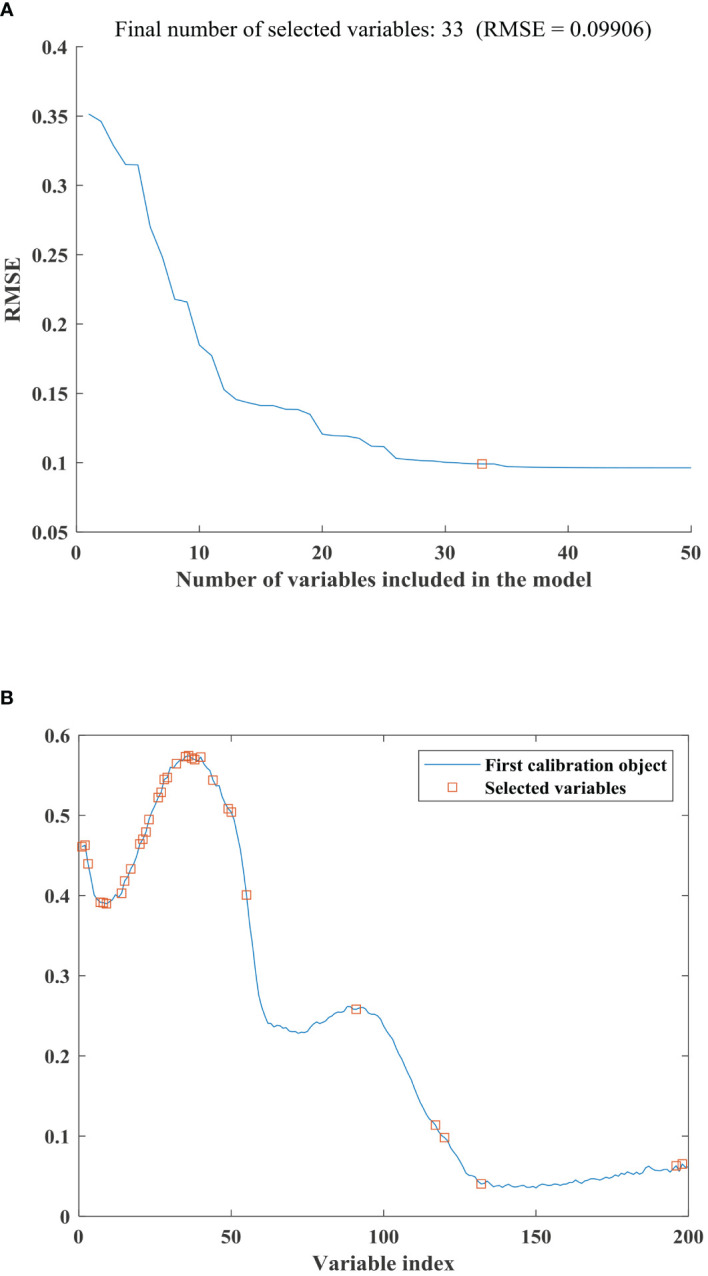
**(A)** The RMSE curve for selecting the number of spectral features using the SPA algorithm. **(B)** Distribution diagram of spectral features selected using the SPA algorithm.

Using SPA for feature extraction on the full spectrum, **Figure 5A** displayed the RMSE plot of the spectral feature extraction by SPA, where empty squares indicated the final number of selected variables. It can be observed that as the number of selected variables increases from 1 to 20, the RMSE curve shows a rapid decline. With a further increase in the number of selected variables, the trend of the RMSE curve becomes relatively stable. When the number of selected variables reached 33, the RMSE reached its optimal value for classification. Improvements in RMSE tend to decrease when the number of features exceeds 33, suggesting that adding more features may not significantly enhance classification performance. Therefore, choosing 33 features balances accuracy while minimizing the feature count. Additionally, fewer features typically imply a simpler model that is easier to interpret and understand. **Figure 5B** depicted the distribution of the selected variables (empty squares) corresponding to the full spectrum. The wavelengths corresponding to the 33 selected features were 953nm, 956nm, 960nm, 963nm, 967nm, 970nm, 974nm, 977nm, 981nm, 998nm, 1001nm, 1008nm, 1019nm, 1022nm, 1026nm, 1029nm, 1040nm, 1060nm, 1071nm, 1074nm, 1081nm, 1088nm, 1102nm, 1120nm, 1123nm, 1141nm, 1266nm, 1358nm, 1368nm, 1411nm, 1638nm, 1645nm and 1648nm.

### 3.3 Image feature analysis

According to the texture characteristics of the blueberry image, the GLCM was generated using four directions (0°, 45°, 90°, or 135°). Four statistical measures (contrast, energy, entropy, and homogeneity) were applied in each direction to extract features, resulting in 16 feature values. Subsequently, the mean value, standard deviation, and variance of these four statistical measures were calculated to generate 12 feature values, totaling 28 feature values describing a blueberry image.

### 3.4 Models results analysis

Based on full spectrum, feature-extracted spectra, image features, and incorporating feature fusion (normalization of spectral and image information), the evaluation metrics of the SVM models, the RF models and the PLS-DA models (accuracy, precision, recall, and *F*1-score) were shown in **Tables 3**–**5**, respectively.

**Table 3:** Comparison of the SVM models based on different category of features.

Feature category	Feature selection method	Number of features	Accuracy (%)	Precision (%)	Recall (%)	*F*1-score (%)
Spectral	Full spectrum	200	88.75	90.83	87.20	88.98
	SPA	33	89.58	92.50	87.40	89.88
Image	GLCM	28	56.25	75.00	54.55	63.16
Fusion	Full spectrum & GLCM	228	90.83	92.50	89.52	90.99
	SPA & GLCM	61	**92.50**	**93.33**	**91.80**	**92.56**

**Table 4:** Comparison of the RF models based on different category of features.

Feature category	Feature selection method	Number of features	Accuracy (%)	Precision (%)	Recall (%)	*F*1-score (%)
Spectral	Full spectrum	200	87.50	88.33	86.89	87.60
	SPA	33	88.33	89.16	87.70	88.42
Image	GLCM	28	56.67	77.50	54.71	64.14
Fusion	Full spectrum & GLCM	228	88.75	90.83	87.20	88.98
	SPA & GLCM	61	90.00	91.67	88.71	90.17

**Table 5:** Comparison of the PLS-DA models based on different category of features.

Feature category	Feature selection method	Number of features	Accuracy (%)	Precision (%)	Recall (%)	*F*1-score (%)
Spectral	Full spectrum	200	85.83	90.00	90.00	90.00
	SPA	33	87.92	91.67	92.44	92.05
Image	GLCM	28	54.58	74.17	53.29	62.02
Fusion	Full spectrum & GLCM	228	89.17	92.50	86.72	89.52
	SPA & GLCM	61	90.83	95.00	87.69	91.20

The settings for SVM model hyperparameters *c* and *g* were both default values.

The results in **Table 3** indicated that, for spectral features, the model achieved an accuracy of 88.75%, a precision of 90.83%, a recall of 87.20%, and an *F*1-score of 88.98% when using the full spectrum. When using the SPA method, the accuracy improved slightly to 89.58%, and the other metrics also increased accordingly. For image features, the performance was relatively poor when using the GLCM method, showing an accuracy of only 56.25%, a precision of 75.00%, a recall of 54.55%, and an *F*1-score of 63.16%. For fusion features, the use of Full-spectrum & GLCM demonstrated good performance across all metrics, with an *F*1-score reaching 90.99%. The best performance was achieved with the SPA & GLCM method, with an accuracy of 92.50% and the highest *F*1-score at 92.56%.

The results of **Tables 4** and **5** exhibited similar conclusions to those of **Table 3**. Combining the results from **Tables 3**–**5**, it was evident that spectral features performed well across all models, particularly when using the SPA method, which further enhanced model performance. Image features performed poorly across all models, suggesting that GLCM features may contribute limitedly to classification tasks. Fusion features, combining spectral and image characteristics, generally improved overall model performance, especially when using the SPA & GLCM method, achieving optimal results. When comparing the performance of different models using the same feature selection methods, the SVM model slightly outperformed RF and PLS-DA. The SVM model that utilized SPA & GLCM information fusion achieved the highest recognition accuracy, reaching 92.50% in the test set. However, an accuracy of 92.50% falls short of meeting the requirements of early detection of post-harvest blueberry damage. Therefore, this study attempted multiple strategies to enhance and optimize the hyperparameters *c* and *g* of the SVM model, with the aim of further enhancing the recognition accuracy of the spectral and image fusion model.

### 3.5 Multiple strategies for improving the BWO parameter analysis

To validate the effectiveness of the proposed method in this study, before optimization, after optimization, and after multiple strategy improvements, the recognition results of the SVM models based on spectral and image fusion were shown in **Table 6**.

**Table 6:** The recognition results before and after optimizing the SVM model based on spectral and image fusion.

SVM	Model hyperparameters		Accuracy (%)	Precision (%)	Recall (%)	*F*1-score (%)
	*c*	*g*				
Unoptimized (Baseline)	Default	Default	92.50	93.33	91.80	92.56
BWO	20.1626	0.2387	94.17	95.83	92.74	94.26
Multi-strategy improved BWO	18.4812	0.2198	**95.00**	**96.67**	**93.55**	**95.08**

The bold text indicates the best classification results.

During the optimization process, the number of iterations was set to 100, and the initial population size was 50. The final hyperparameters were the averages of 30 independent runs of the two optimization algorithms.

The results depicted in **Table 6** highlight the beneficial impact of the BWO algorithm on fine-tuning the hyperparameters of the SVM model. Notably, the BWO algorithm demonstrated significant improvement through multiple strategy enhancements, achieving the highest classification accuracies of 95.00% in the test sets. This increase amounts to 2.50% compared to the baseline performance. Compared to the classic BWO algorithm, this increase was only 0.83%. The possible reasons for such a minor difference might be that the classic BWO algorithm introduced a balancing factor and Lévy flight strategy, which enhanced the algorithm’s performance during the optimization process. These findings indicate that the multi-strategy improved BWO algorithm holds significant promise for parameter optimization in the SVM model.

## 4 Discussion

In light of the above results, this study made the following three discussions:

(1) The classification accuracy of models established based on spectral features extracted using SPA exceeded that of using the full spectrum. One possible reason could be the utilization of SPA to extract the most discriminative features from the spectral information. Such features could assist researchers in comprehending the meaning and underlying patterns of the data, thereby facilitating a better understanding of the attributes and states of the detected objects. Through feature extraction, the data dimensionality was reduced, redundant information was minimized, and the classification accuracy was improved.

The classification model established based on image features extracted from GLCM had a low accuracy. Possible reasons were that early collision damage had a less pronounced effect on the color change of fruit skin, especially for fruits with darker skin color, such as blueberries. The extracted image features of sound and damaged blueberries were quite similar, resulting in the model’s classification accuracy being diminished.

The classification accuracy was improved through the integration of spectral and image information, outperforming the utilization of singular information (either spectral or image). Possible reasons were that high spectral images and spectral information had different feature representations, and their features complemented each other in some aspects. By integrating these two types of information, a better understanding of the spatial and spectral distribution characteristics of the samples could be achieved, leveraging their advantages to enhance the accuracy of the model’s classification.

(2) The possible reasons why SVM outperformed RF and PLS-DA included that SVM classified data by identifying an optimal hyperplane to maximize inter-class separation. It was especially proficient in managing high-dimensional feature spaces and linearly inseparable situations by using kernel methods (such as the RBF kernel) to map data into higher-dimensional spaces, thereby identifying superior classification boundaries. RF, as an ensemble method based on multiple decision trees, was adept at managing high-dimensional features and diverse data. However, the splitting rules of decision trees might encounter difficulties when dealing with highly correlated or redundant features, potentially affecting overall performance. PLS-DA, maximizing the covariance between predictors and response variables through linear combinations, was well-suited for data with linear relationships. In cases of significant nonlinear relationships, its performance might not have been comparable to that of SVM.

Additionally, SVM combined SPA and GLCM feature selection methods, which likely reduced feature redundancy, thereby enhancing the robustness and classification capability of the model. Notably, the SPA method demonstrated excellence in spectral feature selection, effectively extracting key features that contributed to classification.

(3) Based on the multi-strategy enhanced BWO optimization SVM model, compared to the baseline and BWO-optimized SVM models, it attained a higher classification accuracy. One possible explanation is that BWO employed the method of randomly generating the initial population, resulting in an uneven distribution of the population, thereby impacting the convergence speed of the algorithm. The good point set provided an effective approach for selecting points uniformly. It facilitated a more uniform distribution of points in the search space, thereby enhancing global search efficiency. Based on genetic algorithms, Yuan et al. (2022) introduced a good point set to globally optimize the path coverage of unmanned aerial vehicles. They pointed out that the good point set generated a higher quality initial population compared to the randomly distributed algorithm. These high-quality populations were more likely to obtain the global optimal solution. Introducing the elite pool strategy enhanced population diversity, thereby mitigating the drawbacks associated with relying solely on a single best individual for guidance. Liu et al. (2024) proposed an improved arithmetic optimization algorithm combined with a hybrid elite pool strategy. They pointed out that integrating the elite pool strategy into the metaheuristic algorithm enhanced the diversity of the search process and improved the performance of the algorithm across various datasets. As a result, the algorithm’s capability to escape local optima was strengthened. Introducing the adaptive Lévy step size strategy addressed the fluctuating need for expected Lévy step sizes at different algorithmic stages, thereby enhancing the algorithm’s ability to explore the solution space. Additionally, the introduction of the spiral search strategy extensively utilized regional information, thereby enhancing the algorithm’s capability for local search. Golden-SA incorporated the golden ratio coefficient into its position updating process, enabling the algorithm to thoroughly explore areas capable of generating excellent solutions in each iteration. This acceleration improved the algorithm’s convergence speed, enabling it to avoid local optima.

Compared to the baseline model and the classic BWO algorithm-optimized model, the accuracy improved by 2.5% and 0.83%, respectively. The slight improvement suggests that the initial performance of the SVM model is already close to optimal, and further enhancements require more sophisticated optimization techniques. This also implies that while the BWO and its multi-strategy improvements have enhanced model performance, the base model was already well-tuned. Although the increments are minor, these improvements can still be significant in practical applications. In quality control processes, a 1-2% increase in accuracy can substantially reduce the defect rate.

## 5 Conclusions

The study explored the feasibility of detecting early mechanical collision damage in blueberries using a multi-strategy improved BWO optimization SVM model. Specifically, we analyzed the average relative reflectance curves of sound and damaged samples within the range of 950nm to 1650nm. Spectral features were extracted using SPA, while image features were extracted using GLCM. The results indicated that the classification accuracy of the model based on feature fusion was higher than that of single features, whether spectral or image. Then, based on the feature fusion model, attempts were made to utilize a multi-strategy improved BWO algorithm that integrates good point set, elite pool strategy, adaptive Lévy strategy, spiral search strategy, and Golden-SA to optimize the hyperparameters of the SVM model. The results demonstrated that the improved BWO algorithm significantly enhanced the performance of the SVM model. The classification accuracies of the test set was 95.00%. Overall, the combination of spectral and image fusion models with improved optimization techniques provides a powerful approach for enhancing early damage detection in blueberries. Due to the dark color of blueberries, it is difficult to visually identify early mechanical damage. The method proposed in this study has yielded satisfactory results; therefore, we have reason to believe that this method is applicable for detecting mechanical damage in other dark-colored fruits such as blackberries, black grapes, plums, and blackcurrants. This method has significant potential applications in advancing fruit quality assessment and ensuring food safety across the supply chain.

HSI is influenced by varying environmental conditions (such as light intensity and temperature), which can cause inconsistencies in image quality and subsequently affect the model’s detection performance. Therefore, strict control of environmental factors is necessary in practical production applications to ensure detection accuracy. In future research, more diverse blueberry samples will be collected, encompassing different growth stages and environmental conditions, to improve the model’s generalization capability.

## Data Availability

The raw data supporting the conclusions of this article will be made available by the authors, without undue reservation.
